# Acute Copper Toxicity Displays a Nonmonotonic Relationship with Age Across the Medaka (*Oryzias latipes*) Life Span

**DOI:** 10.1002/etc.5481

**Published:** 2022-10-25

**Authors:** Marilyn W. Mason, Benjamin B. Parrott

**Affiliations:** ^1^ Savannah River Ecology Laboratory University of Georgia Aiken South Carolina USA; ^2^ Eugene P. Odum School of Ecology University of Georgia Athens Georgia USA

**Keywords:** Aquatic toxicology, contaminants, copper, life span, teleost

## Abstract

The ability of an organism to cope with environmental stressors varies across the life span because of developmental stage–specific responses and age‐related functional declines. In the present study, we examined the effect of age on acute copper toxicity in Japanese medaka (*Oryzias latipes*). We first determined the median lethal concentration (LC50) at 96 h for embryos, 7‐day‐old fry, and 6‐month‐old medaka. Embryos were exposed to 0, 15, 30, 60, 125, 250, and 500 ppb CuSO_4_ through hatching. Fry were exposed to 0, 20, 50, 75, 100, 150, 250, and 500 ppb CuSO_4_ for 96 h. Adult fish were exposed to 0, 100, 150, 200, 250, and 300 ppb CuSO_4_ for 96 h. The 96‐h LC50 was 804 ppb for embryos, 262 ppb for embryonically exposed larvae, 60.3 ppb for 7‐day‐old fry, and 226 ppb for adults. We then challenged cohorts of fish aged 2, 3, 5, 6, 7, 8, 9, 10, 11, 13, 14, 15, and 16 months with a 225‐ppb CuSO_4_ exposure to determine the acute toxicity across the life span. The fish exhibited a bimodal tolerance to copper, with tolerance peaking in 2‐ and 3‐month‐old fish and again at 10 and 11 months of age. Our data demonstrate that copper sensitivity is dynamic throughout the medaka life span and may be influenced by trade‐offs with reproduction. *Environ Toxicol Chem* 2022;41:2999–3006. © 2022 The Authors. *Environmental Toxicology and Chemistry* published by Wiley Periodicals LLC on behalf of SETAC.

## INTRODUCTION

Anthropogenic activities have led to increasing concentrations of naturally occurring metals in aquatic ecosystems around the world (Zhou et al., 2020). The consequences of these elevated concentrations range from mortality and alterations to life‐history traits at the individual level to population collapse and loss of ecological functions (Sheehan, 1984). In some cases, metals essential for biological functions can accumulate to toxic levels, particularly in aquatic habitats where atmospheric deposition, runoff, and industrial effluent can contribute metals from both remote and point sources to aquatic sinks (Rehman et al., 2019). Copper is of particular concern because it is second only to mercury in metals reported to contribute to poor water quality (Reiley, 2007). Copper can catalyze redox reactions, contributing to its biological activity in numerous enzymes, such as cytochrome *c* oxidase, a vital component for aerobic metabolism (Linder & Hazegh‐Azam, 1996). However, the redox potential of copper also contributes to its toxicity, leading to the production of reactive oxygen species that cause cellular and DNA damage (Craig et al., 2007). The homeostatic control systems that regulate copper in aquatic organisms are adapted to the naturally low levels of 0.2–30 µg L^−1^ found in surface water and can become overwhelmed by copper concentrations in compromised habitats, which can reach 200 mg L^−1^ in areas of active mining (US Environmental Protection Agency [USEPA], 1984). Copper's utility and attendant economic importance have led to a cumulative world production that is approximately 25 times greater than historic levels (Han et al., 2002). The increase in the extraction, and ultimately the environmental dispersion, of copper makes it vitally important to understand the impact copper is having on aquatic systems and the organisms that inhabit them.

In addition to experiencing greater risk of environmental exposure to elevated copper levels than terrestrial organisms, aquatic species are more sensitive to copper toxicity. The highly vascularized gills that most fish use for respiration can account for more than half of an individual's exposed surface area and provide not only gas exchange with the surrounding water but also nutrient ion uptake (Laurent & Perry, 1991). Copper enters the gills through “ionic mimicry,” competing with sodium ions for uptake through an apical Na^+^ channel (Grosell & Wood, 2002). The resulting reactive oxygen species subsequently damage the gills, causing respiratory distress, disruption of the osmoregulatory system, and, ultimately, cardiac collapse and death (Cerqueira & Fernandes, 2002). Even when such exposure does not prove fatal for an individual, it can have long‐lasting repercussions for the organism's overall fitness and a population's viability in contaminated habitats. For example, chronic exposure reduces individual fitness, inhibiting growth, immune function, and reproductive output in many species (Buckley et al., 1982; Choi et al., 2018; Mason et al., 2022; Pickering et al., 1977).

The outcomes of copper exposure vary greatly depending on the life stage of the organism. Copper exposures during development affect embryonic survival, hatching rate, and lateral line development in zebrafish (*Danio rerio*; Johnson et al., 2007). Japanese medaka (*Oryzias latipes*) embryos incubated in contact with copper‐contaminated sediments exhibit skeletal and cardiovascular developmental abnormalities and DNA damage (Barjhoux et al., 2012). Such DNA damage has the potential to influence the trajectory of biological aging, with implications for altered life history and reduced longevity. In adult fathead minnows (*Pimephales promelas*), prespawning exposure to 37 µg L^−1^ significantly reduced egg production (Pickering et al., 1977), and chronic copper exposures to concentrations from 5 to 20 µg L^−1^ reduced survival and altered energy metabolism in the killifish *Poecilia vivipara* (Abou Anni et al., 2019). Although earlier studies found embryos to be less sensitive than larvae (Scott et al., 1976), more recent evidence indicates that the chorion sequesters copper before it reaches the embryo and might serve a protective role in the context of copper exposure. However, the copper that does reach the embryo causes developmental aberrations and reduces survival (Wang et al., 2020). Many studies on copper toxicity in fish focus on specific time points in the life span, particularly the early life stages; yet, how sensitivity to acute copper exposure changes with age in fish is not fully resolved.

Japanese medaka are small fish native to slow‐moving bodies of water in Japan, China, and Korea and are tolerant to a broad range of temperature, pH, and salinity (Shima & Mitani, 2004). Medaka fish can be induced to breed year‐round in the laboratory, and eggs remain attached to the female for several hours and can easily be collected directly from the female (Padilla et al., 2009). The predictability of spawning times, a transparent chorion, and well‐characterized development allow for accurate investigation of the influence of development and age on toxicological responses (Iwamatsu, 2004). In the present study we investigated the acute toxicity of copper sulfate in Japanese medaka as embryos, larvae, and adults. Medaka were considered to be embryos from fertilization until hatching. Adult and larval ages were calculated from hatching. We established the 96‐h median lethal concentration (LC50) for these stages. We then further challenged 13 cohorts of medaka ranging from 2 to 16 months of age with a 225‐ppb CuSO_4_ dose for 168 h to evaluate how medaka sensitivity to acute copper exposure is affected by organismal age.

## MATERIALS AND METHODS

### Fish husbandry

The protocols in the present study were approved by the Institutional Animal Care and Use Committee of the University of Georgia (no. A2019 04‐033‐Y1‐A0). A breeding colony of medaka originating from Aquatic Research Organisms was established at the Savannah River Ecology Laboratory. Fish were maintained in a ZHAB Duo recirculating system (Pentair AES) at 27 °C on a 14: 10‐h photoperiod. The system was supplied with deionized water buffered with Crystal Sea® Marinemix artificial sea salt to a conductivity of 350 µS to provide essential trace elements, minerals, and electrolytes to aid in osmoregulation and maintain fish health. Crystal Sea contains a mix of major, minor, and trace elements, including chloride, magnesium, sodium, potassium, calcium, and bicarbonate among others. Sodium bicarbonate was added to maintain a pH of 7.2. The general hardness of the system was 50 ppm, and the carbonate hardness was 25 ppm (Table 1). Fish of all ages were fed ground Tetramin® Tropical Flakes twice daily and brine shrimp nauplii (*Artemia* sp.; Brine Shrimp Direct) once daily. Embryos were collected in the morning and incubated in embryo rearing media (40 mg L^−1^ CaCl_2_ × 2H_2_O, 1 g L^−1^ NaCl, 30 mg L^−1^ KCl, 163 mg L^−1^ MgSO_4_ × 7H_2_O, 1 mg L^−1^ Kordon® Methylene Blue in deionized water) until hatching. The embryo rearing solution had a general hardness of 100 ppm and a carbonate hardness of 25 ppm. Fry were transferred by pipette to 1.5‐L containers of clean water from the recirculating system and reared until large enough to put into the recirculating system. A separate colony of medaka from the OK‐Cab MT480 strain originating from NBRP Medaka was established and maintained under the same conditions as the main breeding colony.

**Table 1:** Water parameters for buffered deionized water and embryo rearing solution

Parameter	Buffered DI water	Embryo rearing solution
pH	7.2	7.1
GH (ppm)	50	100
KH (ppm)	25	25
Ammonia (ppm)	0	0
Nitrite (ppm)	0	0
Nitrate (ppm)	0	0

DI = deionized; GH = general hardness; KH = carbonate hardness.

### Copper concentrations

To validate our copper dosing concentrations across different temperatures (27 °C, 30 °C, and 33 °C) and the stability of the doses over time, we dosed 50‐ml aliquots of system water to 10 and 100 ppb CuSO_4_ (Sigma‐Aldrich) to match experimental conditions. Temperature for all subsequent dosing was maintained at approximately 27 °C. Duplicate 10‐ml samples were collected at 0‐, 1‐, 24‐, 48‐, and 72‐h time points for analysis (Supporting Information, Figure S1 and Table S1). Samples were acidified with 0.2 ml HNO_3_ prior to analysis. Copper ion concentrations were analyzed following USEPA Method 6020A for inductively coupled plasma‐mass spectroscopy (Nexlon 300X ICP‐MS; Perkin‐Elmer) on diluted samples (USEPA, 2007). Scandium 45 and indium 115 were used as internal standards. The method detection limit for copper ion was 0.0163 ppb. All samples were above the method detection limit. The mean percentage of recovery for copper 63 in CRM1640a Natural Water Standard used as quality control was 101%. Data were not corrected for percentage of recovery.

### Embryo exposures

Dosing of CuSO_4_ was performed as in our previous study (Mason et al., 2022). Briefly, medaka embryos were collected within 1 h of the start of the light cycle and staged under a dissecting scope. Clutches of embryos that were Stage 6 or less (within 3 h of fertilization) were divided among 50‐ml containers of prepared embryo rearing media dosed with CuSO_4_ to 0‐, 15‐, 30‐, 60‐, 125‐, 250‐, and 500‐ppb concentrations. There were three replicates with 10 embryos per treatment. The embryos were checked daily using a binocular dissecting scope, and dead eggs were recorded and removed. Embryos were checked for abnormal development and considered dead if coagulation or a lack of heartbeat was detected. Hatched fry were recorded daily and moved to a 50‐ml container of deionized water buffered to match the system conditions and dosed with the same concentration of CuSO_4_ (Sigma‐Aldrich) as the embryos. The fry were maintained in these conditions for 96 h with one water change at 48 h, then euthanized with an overdose of tricaine methanesulfonate. Mortalities were recorded daily. Fry were considered dead when no gill movement or heartbeat could be detected when viewed under a binocular dissecting scope.

### Fry exposures

Fish were reared for 6–8 days in standard conditions. Containers with 500 ml of deionized water buffered to match the rearing conditions and dosed with copper sulfate were prepared in concentrations of 0, 20, 50, 75, 100, 150, 250, and 500 ppb for four replicates. The first replicate did not include the 50‐ and 75‐ppb doses. Fish were randomly selected and transferred by pipette to the dosing containers in groups of 10. The dosed water was changed every 48 h for 96 h. The fish were checked daily under a binocular dissecting scope, and mortalities were recorded and removed. Fry were considered dead when no gill movement or heartbeat could be detected.

### Adult exposures

Fish were reared to 6 months of age in standard conditions. One week prior to copper exposure, the fish were sorted by sex (determined by examining sexually dimorphic dorsal and anal fin morphology) and transferred to tanks with deionized water buffered to match the system conditions. Containers with 500 ml of deionized water buffered to match system conditions were dosed with CuSO_4_ to concentrations of 0, 100, 150, 200, 250, and 300 ppb for five replicates. Fish were randomly divided into groups of three males and three females and transferred into the dosing containers. The dosed water was replaced every 48 h for 96 h. The fish were checked daily, and mortalities were recorded and removed. Fish were considered dead when no gill movement could be detected.

### Life‐span exposures

Cohorts of Ok‐Cab medaka were collected at the beginning of each month and maintained under standard conditions. A range of 13 cohorts aged 2–16 months were divided into groups and transferred into 500‐ml containers of buffered, deionized water dosed with 0 or 225 ppb CuSO_4_ for one to three replicates (Table 2). The water was replaced every 48 h for 168 h. The fish were checked daily, and mortalities were recorded, sexed, and removed.

**Table 2:** Number of fish for each age cohort in each replicate dosed with 225 ppb CuSO_4_

Age (months)	0 ppb	225 ppb Rep 1	225 ppb Rep 2	225 ppb Rep 3
2	5	5	5	
3	5	5		
5	5	5	5	5
6	4	4	4	
7	4	4	4	
8	2	3		
9	2	3		
10	3	3		
11	4	4	4	
13	5	5		
14	5	5	5	5
15	4	4	4	
16	3	3	3	

Rep = replicate.

### Statistical analysis

The LC50 for embryos, hatched fry, 7‐day‐old, and 6‐month‐old fish were calculated using probit analysis based on Finney's method in Excel (Srinivasan, 2004). Comparisons of hatching status in embryos were made with a logistical regression in R (Ver 4.0.4; R, 2021). Male versus female mortality in the adult LC50 cohort were compared using a two‐way analysis of variance (ANOVA) performed in R. Comparisons of survival time for the age cohorts were made using a two‐way ANOVA performed in R. Differences among groups were compared with a Tukey honestly significant difference test. Proportions of fish surviving at 96 h were compared using a χ^2^ analysis in R.

## RESULTS

### Embryo exposures

**Figure 1:**
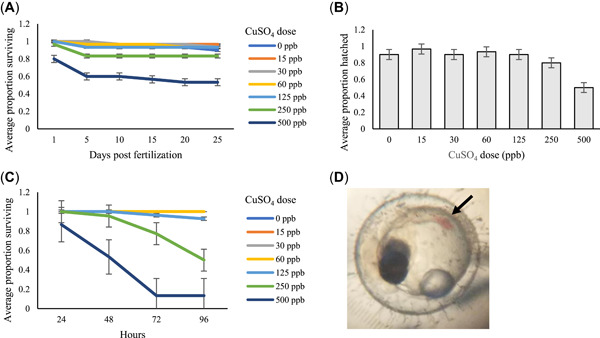
Medaka embryos (three replicates of 10 embryos each) exposed to CuSO_4_ during development. (**A**) Average proportion of embryos surviving each day of copper exposure. (**B**) Average proportion of embryos hatched; 500 ppb CuSO_4_ significantly reduced hatch success (*z* = −3.1, *p* = 0.002). (**C**) Average proportion of fry surviving over 96 h of CuSO_4_ exposure posthatch. (**D**) Malformed embryo exposed to 125 ppb missing caudal region and showing blood pooling (arrow). Error bars represent standard error.

For embryos, exposure began within 3 h of fertilization and lasted until hatching. Most of the embryos hatched between 11 and 16 days. During the first 96 h of exposure, the LC50 for embryo medaka was 804 ppb, with a lower limit of 374 ppb and an upper limit of 1730 ppb (χ^2^ = 1.03; Figure 1A). Most of the embryo mortality occurred within the first 96 h of exposure. Only the 500‐ppb dose significantly affected hatch success (Figure 1B; *z* = −3.1, *p* = 0.002). There was no significant effect of dose on time to hatch. Fry exposed to CuSO_4_ as embryos were maintained at the same dose for an additional 96 h posthatch to determine their LC50. The LC50 for the hatched fry was 324 ppb at 72 h, with a lower limit of 266 ppb and an upper limit of 396 ppb (χ^2^ = 0.92; Figure 1C). At 96 h the LC50 was 262 ppb, with a lower limit of 214 ppb and an upper limit of 321 ppb. In addition, deformities including small eyes, pooling of blood, and an absence of the tail region were observed among embryos reared in 30‐ppb CuSO_4_ concentration and higher (Figure 1D). These sublethal effects were not included as part of the mortality endpoints. Hatched fry exposed to copper exhibited a range of behavioral abnormalities, including jerking movements and lethargy at all concentrations.

### Fry LC50

**Figure 2:**
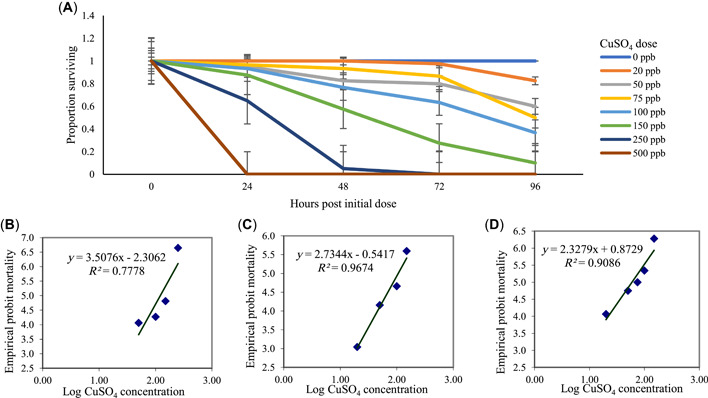
Exposures to CuSO_4_ to determine the median lethal concentration (LC50) of 7‐day‐old medaka fry (four replicates of 10 fry each). (**A**) Average proportion of fry surviving over 96 h. Error bars represent standard error. (**B**) Probit analysis of fry mortality at 48 h. The LC50 at 48 h was 130 ppb (χ^2^ = 15.2). (**C**) Probit analysis of fry mortality at 72 h. The LC50 at 72 h was 105 ppb (χ^2^ = 2.15). (**D**) Probit analysis of fry mortality at 96 h. The LC50 at 96 h was 60.3 ppb (χ^2^ = 3.79).

The proportion of surviving 1‐week‐old fry, which had no prior exposure to copper, was tracked for 96 h (Figure 2A). The LC50 at 48 h was 130 ppb, with a lower limit of 110 ppb and an upper limit of 153 ppb (χ^2^ = 15.217; Figure 2B). The LC50 at 72 h was 105 ppb, with a lower limit of 85.1 ppb and an upper limit of 129 ppb (χ^2^ = 2.15; Figure 2C). The LC50 at 96 h was 60.3 ppb, with a lower limit of 49.3 ppb and an upper limit of 73.8 ppb (χ^2^ = 3.79; Figure 2D).

### Adult LC50

**Figure 3:**
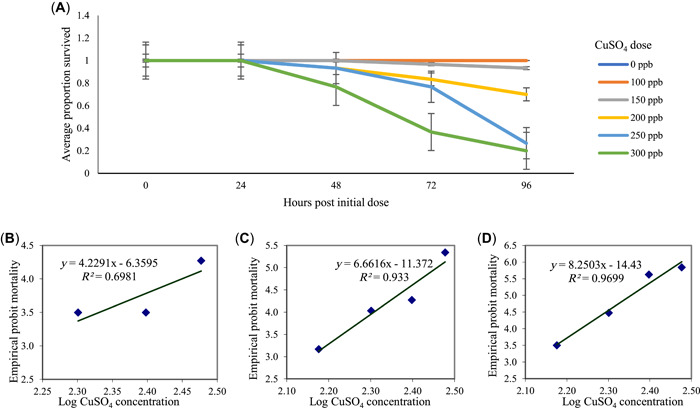
Exposures to CuSO_4_ to determine the median lethal concentration (LC50) of 6‐month‐old adult medaka (five replicates of six fish each). (**A**) Average proportion of fish surviving over 96 h. Error bars represent standard error. (**B**) Probit analysis of fish mortality at 48 h. The LC50 at 48 h was 446 ppb (χ^2^ = 1.16). (**C**) Probit analysis of fish mortality at 72 h. The LC50 at 72 h was 285 ppb (χ^2^ = 2.56). (**D**) Probit analysis of fish mortality at 96 h. The LC50 at 96 h was 226 ppb (χ^2^ = 1.69).

The proportion of surviving 6‐month‐old fish was tracked for 96 h (Figure 3A). The LC50 at 48 h was 446 ppb, with a lower limit of 240 ppb and an upper limit of 828 ppb (χ^2^ = 1.16; Figure 3B). The LC50 at 72 h was 285 ppb, with a lower limit of 225 ppb and an upper limit of 318 ppb (χ^2^ = 2.56; Figure 3C). The LC50 at 96 h was 226 ppb, with a lower limit of 210 ppb and an upper limit of 243 ppb (χ^2^ = 1.69; Figure 3D). There was no significant difference in survival between the sexes (*F* = 0.076, *p* = 0.78) or any interaction between the copper dose and sex (*F* = 1.84, *p* = 0.12).

### Life‐span exposures

**Figure 4:**
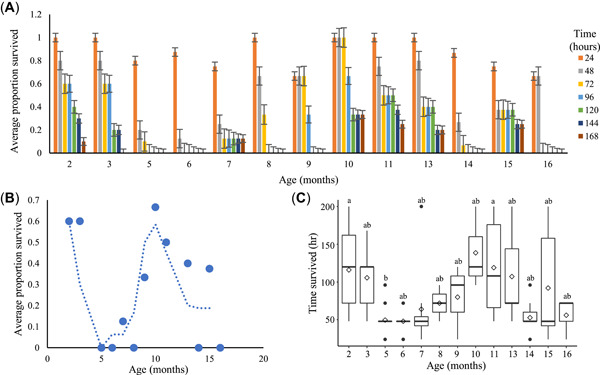
Acute exposure to 225 ppb CuSO_4_ for medaka of different ages. (**A**) Average proportion of fish surviving for each age cohort. Error bars represent standard error. (**B**) Average proportion of fish surviving across the 168‐h exposure. (**C**) Hours of survival for age cohorts across the 168‐h exposure. Fish surviving past the 168‐h point survived to the end of the exposure. Fish age (months posthatch) significantly affected survival time (*F* = 3.19, *p* = 0.0009). Horizontal bars represent median, diamonds represent mean, and letters indicate statistically significant differences among groups.

There was a significant difference in the length of survival time among different aged fish (*F* = 3.19, *p* = 0.0009; Figure 4A). At 96 h post–initial exposure, there was a significant difference in survival among the age groups (χ^2^ = 24.9, degrees of freedom = 12, *p* = 0.016; Figure 4B). The youngest cohorts (2 and 3 months old) and cohorts in the middle of the age range (9, 10, and 11 months) displayed a longer time of survival and a higher proportion of individuals surviving to the end of the experiment (Figure 4C). Sex did not have a significant effect on survival.

## DISCUSSION

There are many factors that influence an individual's susceptibility to toxic contaminants, from species‐specific tolerance to developmental sensitivities. Embryos exposed to copper are generally less sensitive than other life stages, likely because of the protection afforded by the chorion (Wang et al., 2020). Wang et al. (2020) demonstrated in a related medaka species, *Oryzias melastigma*, that >90% of the copper taken up by the embryos accumulated in the chorion. In the present study, only the highest dose of 500 ppb CuSO_4_ significantly affected overall hatch success, although critical morphological deformities were observed in a few embryos at doses as low as 30 ppb. Fry exposed to CuSO_4_ as embryos were much less sensitive than naive 7‐day‐old fry, indicating that embryonic exposure may induce acclimation to copper. However, the 7‐day‐old fry may also have been at a more sensitive developmental point, and studies in other species have noted less sensitivity to copper in newly hatched fish compared to fry just a few days older. For example, McNulty et al. (1994) found that topsmelt (*Atherinops affinis*) larvae were less sensitive to CuCl_2_ from hatching through day 5 posthatch compared to fish that were 7 days or older at the time of exposure. This age‐dependent sensitivity observed in topsmelt correlates to increasing gill and cutaneous surface area and suggests that the transition between cutaneous and branchial respiration may increase the amount of metal taken up by the dual forms of respiration and underlie the enhanced sensitivity observed during this period (McNulty et al., 1994). These findings may partly explain the reduced sensitivity in our embryonically exposed fry, but it likely had less of an effect in our system because medaka already possess rudimentary gill rakers at hatch and develop lamellae in as little as 2 days (Leguen, 2018). This rapid development and early branchial respiration would suggest that medaka should display a transition in posthatching copper sensitivity earlier. Additional experiments incorporating fine temporal scales are required to further resolve copper toxicity in early posthatchling fry and attendant physiological and anatomical correlates.

The decrease in acute copper sensitivity occurring between the 7‐day‐old fish and the 6‐month‐old fish in the present study is consistent with acute toxicity reported in other species. Kousar and Javed (2012) found a decrease in copper sensitivity with increasing age in fish aged 90, 120, and 150 days in four different species. However, other studies have demonstrated a conflicting trend. For example, red sea bream (*Pagrus major*) were more sensitive to copper with increasing size from 0.5 to 12.8 g (Furuta et al., 2008), suggesting that life‐stage toxicity of copper might be taxon‐specific. Yet, what underlying mechanisms mediate ontogenetic shifts in sensitivity to copper exposure across different species are unresolved.

Our data on copper toxicity across the medaka life span indicate that acute toxicity is dynamic far beyond the early life stages and cannot be accounted for with measurements of body size or age. The bimodal distribution we report in copper tolerance suggests that other life‐history factors are likely to exert profound effects on the outcome of acute exposures. Medaka typically reach sexual maturity at approximately 2–3 months of age and can be induced to spawn continuously for approximately 3 months until they are exhausted (Leaf et al., 2011). The individuals in the youngest of our life‐span cohorts (2 and 3 months of age) were just prior to or at the beginning of reaching maturity. The fish at the peak reproductive ages of 5 and 6 months were among the least tolerant to copper exposure. This suggests that reproductive investment may play an important part in determining a fish's ability to cope with copper exposure. Copper exposure has been shown to reduce fecundity in fish and decrease the expression of the estrogen receptor gene ER‐α in the livers of female fathead minnows (*Pimephales promelas*; Driessnack et al., 2016). Reproduction can affect metallothionein production in fish and has been associated with an increase in hepatic and serum zinc, indicating a spawning‐specific change in metal homeostasis (Overnell et al., 1987). While reproduction increases the basal expression of metallothionein genes, which should help mitigate the oxidative damage caused by copper, it may suggest that these systems are already under stress during spawning season. The increase in metallothionein production is only seen in females in some species, such as plaice (*Pleuronectes platessa*), whereas in other species, such as the killifish *Fundulus heteroclitus*, it is seen in both sexes (Van Cleef et al., 2000; Overnell et al., 1987). Additional experiments are needed to elucidate the influence of reproductive investment in responses to metal exposure and vice versa. The fish in some of the oldest cohorts (14 and 16 months) also showed a decreased tolerance to copper exposure, possibly due to age‐related decline in their ability to manage copper‐induced stress or repair the resulting damage. The difference in copper sensitivity could be related to the varying metabolic demands of development, growth, and reproduction, as suggested by Hoang et al. (2004) for nickel toxicity in larval and juvenile fathead minnows.

Overall, the present study demonstrates the importance of considering age in determining acute toxicity within a species. We also show the potential of even a transient influx of a contaminant like CuSO_4_ to affect the structure of exposed populations. In medaka, both fry and fish at their reproductive peak are more susceptible to mortality from acute exposures. A significant loss of such individuals could be detrimental to vulnerable populations.

## Supporting information

The Supporting Information is available on the Wiley Online Library at https://doi.org/10.1002/etc.5481.

## Disclaimer

The present study was prepared as an account of work sponsored by an agency of the US government. Neither the US government nor any agency thereof nor any of its employees makes any warranty, express or implied, or assumes any legal liability or responsibility for the accuracy, completeness, or usefulness of any information, apparatus, product, or process disclosed. Reference herein to any specific commercial product, process, or service by trade name, trademark, manufacturer, or otherwise does not necessarily constitute or imply its endorsement, recommendation, or favoring by the US government or any agency thereof. The view and opinions of the authors expressed herein do not necessarily state or reflect those of the US government or any agency thereof.

## Author Contributions Statement

**Marilyn W. Mason**: Methodology; Investigation; Data curation; Formal analysis; Visualization; Writing—original draft. **Benjamin B. Parrott**: Conceptualization; Methodology; Resources; Writing—review & editing; Funding acquisition; Supervision.

## Supporting information

This article includes online‐only Supporting Information.

Supplementary information.

Supplementary information.

Supplementary information.

## Data Availability

The data for the present study are found in the Supplemental Information. Data, associated metadata, and calculation tools are also available from the corresponding author (mwmason@uga.edu).
