# Supplementary material for: Acute Copper Toxicity Displays a Nonmonotonic Relationship with Age Across the Medaka (Oryzias latipes) Life Span
Source: Environ Toxicol Chem. 2022 Oct 25;41(12):2999–3006. doi: 10.1002/etc.5481 (PMC9828168; doi:10.1002/etc.5481)
Supplement: Supplementary file 1 — Supplementary information. [file ETC-41-2999-s001.pdf]

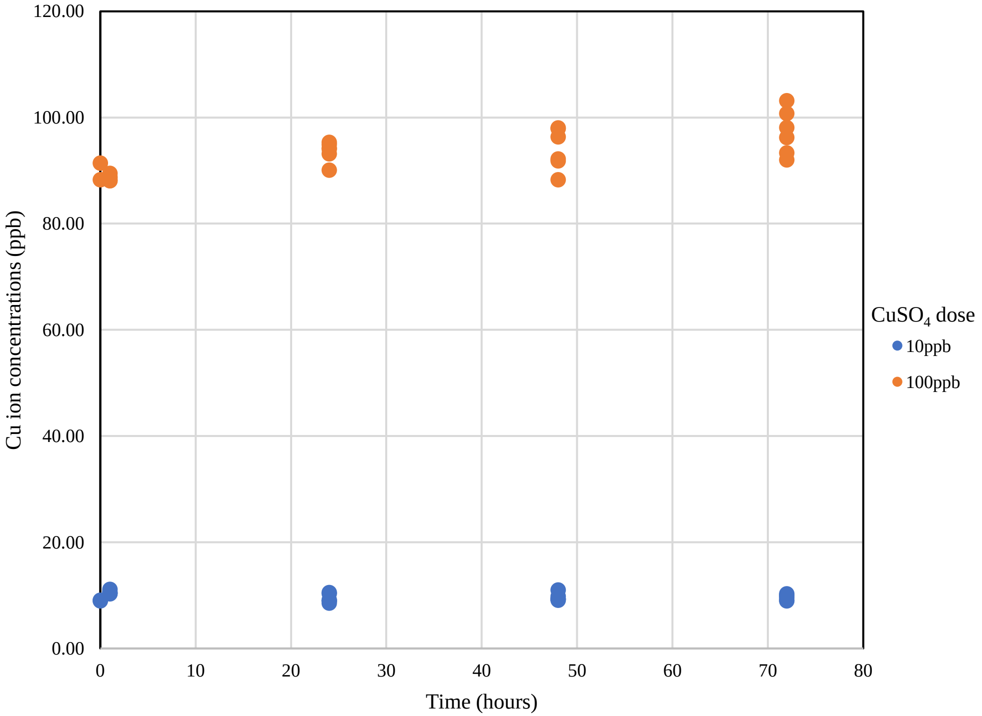

Figure S1. Copper ion concentration validation in system water dosed with CuSO4 over a 3-day period.
