# Supplementary material for: Acute Copper Toxicity Displays a Nonmonotonic Relationship with Age Across the Medaka (Oryzias latipes) Life Span
Source: Environ Toxicol Chem. 2022 Oct 25;41(12):2999–3006. doi: 10.1002/etc.5481 (PMC9828168; doi:10.1002/etc.5481)
Supplement: Supplementary file 2 — Supplementary information. [file ETC-41-2999-s002.docx]

Table S1. Average measured copper ion concentration in water dosed with CuSO_4_ sampled in duplicate across a 3-day period.

| Dosing | 0 hr | 1 hr | 24 hr | 48 hr | 72 hr |
| --- | --- | --- | --- | --- | --- |
| 10 ppb |  |  |  |  |  |
| 27 °C | 9.03 | 10.9 | 10.4 | 10.4 | 9.02 |
| 30 °C |  | 10.4 | 8.76 | 9.20 | 9.77 |
| 33 °C |  | 10.4 | 9.05 | 9.28 | 10.06 |
| 100 ppb |  |  |  |  |  |
| 27 °C | 89.9 | 88.6 | 92.5 | 90.3 | 92.7 |
| 30 °C |  | 88.8 | 93.6 | 98.0 | 98.5 |
| 33 °C |  | 88.4 | 94.8 | 94.1 | 100.6 |
